# An efficient synthesis of indolo[2,3-b]quinoline guanidine derivatives with their in vitro and in vivo study

**DOI:** 10.1007/s00044-017-2028-1

**Published:** 2017-09-05

**Authors:** Katarzyna Sidoryk, Marta Świtalska, Piotr Rózga, Joanna Wietrzyk, Iwona Bujak, Bartłomiej Żerek, Łukasz Kaczmarek, Marcin Cybulski

**Affiliations:** 1 Pharmaceutical Research Institute, 8 Rydygiera St., 01-793 Warszawa, Poland; 2 Institute of Immunology and Experimental Therapy, Polish Academy of Sciences, 12 Weigla St., 53-114 Wrocław, Poland; 3 Adamed Group, Oncology Group, Pieńków 149, 05-152 Czosnów, Poland

**Keywords:** Guanidine group, Indolo[2,3-b]quinoline, Guanidylation, Antiproliferative activity, Anticancer activity

## Abstract

An optimization of the guanidylation process by verifying the efficacy of common guanylation reagents in order to obtain the guanidine derivatives of indolo[2,3-b]quinoline has been performed. As a result, a high-yield procedure using *N*,*N*′-di-Boc-*N*′′-triflylguanidine was applied to synthesize the guanidine derivative of indolo[2,3-b]quinoline **1** in a gram scale for specific in vitro and in vivo biological research. Extensive studies on the antiproliferative activity against eight human tumor cell lines were completed. Compound **1** revealed the highest activity against A549 lung adenocarcinoma and MCF7 breast cancer cell lines. Thus, **1** was evaluated for the in vivo anticancer activity against 4T1 mammary gland carcinoma and KLN205 murine lung carcinoma in mouse models. The anticancer effect was observed in the KLN205 model with a 37% tumor growth inhibition at the 20 mg/kg dose. This anticancer activity of **1** was comparable to that of cyclophosphamide which inhibited murine lung tumor growth in the range of 27–43% at the dose of 100 mg/kg. The biochemistry research after **1** admission, including measurements of blood parameters like alanine aminotransferase, aspartate aminotransferase, lactate dehydrogenase, and urea and creatinine, were also performed.

## Introduction

**Fig. 1:**
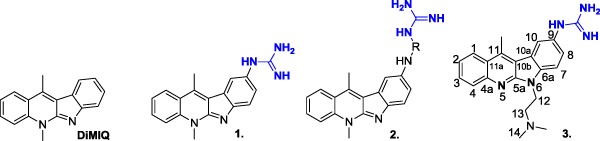
The structures of indolo[2,3-b]quinoline guanidine derivatives, R—amino acid

The guanidine group widely exists in various natural products and pharmaceutically active compounds (Yamamoto et al. 1991; Heys et al. 2000; Snider and Shi 1994; Coffey et al. 2000; Yuan and Williams 1997; Orner and Hamilton 2001). Due to its strong basic properties (pK_a_ 12.5), it actively interacts with the phosphate residues of the minor groove of the DNA helix (Králová et al. 2003). Besides stabilizing the drug-DNA complex, the guanidine group may also improve the delivery of the substances inside cancer cells by increasing their hydrophilic properties and water solubility, while simultaneously decreasing their toxicity (Hau et al. 2002; Liu et al. 2006; Pantos et al. 2008; Chari 2008). Our previous studies have demonstrated that the attachment of a guanidine or guanyl-amino acid chain to the indolo[2,3-b]quinoline core significantly improved its selectivity by increasing the cytotoxic activity against some cancer cell lines, but not against normal cells (Fig. 1) (Sidoryk et al. 2015). The cytotoxic activity of the guanidine-DiMIQ derivative (**1**) was notably higher against cancer cell lines than against normal cells (mice fibroblasts BALB/3T3) (Sidoryk et al. 2015).

Such extremely high selectivity has rarely been observed for standard anticancer drugs. For example, the cytotoxicity of Doxorubicin, widely used in cancer treatment against both cancer and normal cell lines, is comparable. Moreover, the guanidine derivatives of DiMIQ (**1** and **3**) are very effective inducers of apoptosis. It follows that the extreme selectivity of the guanidine-DiMIQ conjugate may result from a new, unique molecular mechanism of action. Also, an important and promising feature of these compounds is the absence of hemolysis (Sidoryk et al. 2015).

Thus **1** and **3** derivatives have been selected for the extensive evaluation of their in vitro antiproliferative activity against several types of cancer cell lines (A549, NCI-H460, HCT116, HT-29, ACHN, HepG2, MCF-7, and A431) and normal cells (human fibroblasts CCD11Lu). In addition, the impact of the compounds on tumor growth was to be investigated in an in vivo mouse model. During our previous studies, it had been experimentally verified that the guanidylation by *N,N*’-Bis(tert-butyloxycarbonyl)-*S*-methylisothiourea in a two-step synthetic sequence allows to obtain samples of guanidine derivatives for biological screening in average yields. However, this approach seemed to be problematic in the upscaling process because of the release of mephitic methyl mercaptan. For that reason, it was necessary to find a new, effective, and reproducible synthetic method which would lead us to obtain **1** and **3** guanidine analogs in a gram scale for pre-clinical tests.

In this paper, we describe the optimization process, a new, improved synthetic strategy for the production of guanidine analogs, and biological in vitro and in vivo characteristics of compounds **1** and **3**.

## Matarials and methods

### Chemistry

#### General

The ^1^H and ^13^C nuclear magnetic resonance (NMR) spectra of all compounds studied were measured in CDCl_3_, dimethyl sulfoxide (DMSO)-D_6_, or D_2_O using Varian-NMR-vnmrs500, Varian-NMR-vnmrs600, and Varian Gemini 200 spectrometers at the temperature of 298 K. Standard experimental conditions and standard Varian programs were used. To assign the structures under consideration, the following 1D and 2D experiments were employed: the 1D selective NOESY and 2D gradient-selected COSY, ^1^H-^13^C heteronuclear single quantum correlation, and ^1^H-^13^C Heteronuclear Multiple Bond Correlation. The ^1^H and ^13^C NMR chemical shifts relate to the tetramethylsilane (for compounds dissolved in CDCl_3_ or DMSO-D_6_) and DSS (for compounds dissolved in D_2_O). The concentration of all solutions used for the measurements was about 10–20 mg of the compounds in 0.6–0.8 cm^3^ of the solvent.

The electrospray ionization-mass spectrometry (ESI-MS) spectra were recorded on a PE Biosystems Mariner mass spectrometer. The progress of the reaction was monitored by thin layer chromatography (TLC) using Merck DC-Alufolien Kieselgel 60 F_254_. The chemicals and solvents were purchased from Fluka Company. Column chromatography was performed on Merck silica gel 60 (230–400 mesh).

The chromatographic analysis was performed using a Shimadzu high performance liquid chromatography (HPLC) system (Shimadzu Corporation, Japan) consisting of a Shimadzu LC-20AB pump, Shimadzu SIL-20AC autosampler, Shimadzu CTO-10AS VP column oven, and Shimadzu SPD-M20A photodiode array detector. The separation of the analyte from potential impurities was achieved using a Kromasil C8 column (150 × 4.6 mm, 3.5 µm, Kromasil) placed in a thermostated column heater at 30 °C. The mobile phases consisting of A (0.1% TFA in water) and B (0.1% TFA in acetonitrile) were used with the gradient mode at the flow rate of 1 ml/min. The samples were prepared at the concentration of about 0.2 mg/ml and they were dissolved in methanol (compound **5**) and water (compound **1**). The injection volume was 10 µl. Ultraviolet (UV) detection at 275 nm was used.

##### *N-*[bis(*tert*-butyloxycarbonyl)guanyl]-*N*-(5,11-dimethyl-5*H*-indolo[2,3-b]quinolin-9-yl)-amine (**5**)

The amino component **4** (1.5 g, 5.75 mM) was added to the solution of DIPEA (2 ml, 11.5 mM) and *N,N*-di-Boc-*N'*′-triflylguanidine (2.47 g, 6.32 mM) in DCM (40 ml). The mixture was stirred for 2 h at room temperature (TLC monitoring, chloroform: methanol 9:1 v/v). Next, the mixture was washed with 2 M aqueous sodium bisulfate (50 ml) and saturated sodium bicarbonate (50 ml). Each aqueous layer was extracted with dichloromethane (2 × 30 ml). The combined organic phases were washed with brine (40 ml), dried (MgSO_4_), filtered, and concentrated under reduced pressure to afford crude compound **5**. The compound was purified by column chromatography (chloroform: methanol 9:1 v/v) to afford a title compound **5** (2.71 g) as an orange solid with a good 94.61% yield. mp 210 °C (decomp.); ^1^H NMR (CDCl_3_, 600 MHz): *δ* = 8.78 (1H, d, *J* = 1.84 Hz, H-10), 8.24–8.21 (1H, m, H-1), 7.79–7.77 (2H, m, H-3, H-4), 7.65 (1H, d, *J* = 8.4 Hz, H-7), 7.61–7.26 (2H, m, H-2, H-8), 4.33 (3H, s, Ar–CH_3_), 3.12 (3H, s, Ar–CH_3_), 1.57 (9H, s, *t*-Bu), 1.52 (9H, s, *t*-Bu); ^13^C NMR (CDCl_3_, 150 MHz): *δ* = 163.5 (C, HNC(NH)_2_), 154.2 (C, 5a), 153.5 (C, NHCO-*t*-Bu), 153.3 (C, NCO-*t*-Bu), 150.0 (C, 6a), 141.5 (C, C-11), 136.5 (C, C-4a), 130.5 (CH, C-3), 129.5 (C, C-10a), 125.8 (CH, C-1), 124.6 (C, C-10b), 124.1(C, C-9), 123.2 (CH, C-8), 122.1 (CH, C-2), 121.5 (C, C-11a), 117.8 (CH, C-10), 116.6 (CH, C-7), 114.7 (CH, C-4), 83.5 (C, *t*-Bu), 79.2 (CH_3_, *t*-Bu), 33.5 (CH_3_, Ar–CH_3_), 28.2 (CH_3_, *t*-Bu), 28.1 (CH_3_, *t*-Bu), 15.1(CH_3_, Ar–CH_3_); HRESIMS *m*/*z*: 504.2611 (C_28_H_34_N_5_O_4_) [M + H]^+^ (calcd. 504.2605). The ^1^H NMR and ^13^C NMR data obtained were in good agreement with that reported in the literature (Sidoryk et al. 2015). HPLC: 94.82%.

##### *N*-guanyl-*N*-(5,11-dimethyl-5*H*-indolo[2,3-b]qinolin-9-yl)-amine dihydrochloride (**1**)

Boc-derivative **5** (2.6 g, 5.16 mM) was treated with TFA (30 ml) and stirred for 24 h (TLC monitoring). The solution was evaporated to dryness, next HCl/CH_3_OH was added and evaporated to dryness. This procedure was repeated three times. The residue was crystallized from ethyl acetate to afford a yellow solid; yield 1.64 g (84%); mp 260 °C(decomp.); ^1^H NMR (D_2_O, 600 MHz): *δ* = 8.16 (1H, dd, *J* = 3.3 Hz, *J* = 24 Hz, H-1), 7.95–7.87 (1H, m, H-3), 7.86–7.84 (2H, m, H-4, H-10), 7.66–7.58 (1H, m, H-2), 7.48 (1H, d, *J* = 23 Hz, H-7), 7.39 (1H, dd, *J* = 5 Hz, *J* = 23 Hz, H-8), 3.92 (3H, s, Ar–CH_3_), 2.76 (3H, s, Ar–CH_3_); ^13^C NMR (D_2_O, 150 MHz): *δ* = 159.3 (C, HNC(NH)_2_), 152.7 (C, C-11), 148.8 (C, C-5a), 140.8 (C, C-6a), 137.4 (C, C-4a), 136.7 (CH, C-3), 132.3 (C, C-9), 130.2 (CH, C-8), 129.1 (CH, C-1), 128.8 (CH, C-2), 125.2 (C, C-11a), 124.1 (CH, C-10), 123.7 (C, C-10a), 120.9 (C, C-10b), 118.6 (CH, C-4), 116.3 (CH, C-7), 38.2 (CH_3_, Ar–CH_3_), 18.2 (CH_3_, Ar–CH_3_); The ^1^H NMR and ^13^C NMR data obtained were in good agreement with that reported in the literature (Sidoryk et al. 2015); HRESIMS *m*/*z*: 304.1562 (C_18_H_18_N_5_) [M + H]^+^ (calcd. 304.1560); anal. calcd. for C_18_H_17_N_5_ × 2HCl: C, 57.45; H, 5.09; N, 18.61; Cl, 18.84 found: C, 57.26; H, 5.18; N, 18.44; Cl, 18.67; HPLC: 97.38%.

##### *N*-[bis(*tert*-butyloxycarbonyl)guanyl]-*N*-[6-(2-dimethylaminoethyl)-11-methyl-6*H*-indolo[2,3-b]quinolin-9-yl]-amine (**7**)

The amino component **6** (200 mg, 0.63 mM) was added to the solution of DIPEA (0.33 ml, 1.89 mM) and *N,N*-di-Boc-*N*′′-triflylguanidine (271.2 mg, 0.693 mM) in DCM (10 ml). The mixture was stirred for 3 h at room temperature (TLC monitoring, chloroform:methanol 9:1 v/v). Next, the mixture was washed with 2 M aqueous sodium bisulfate (15 ml) and saturated sodium bicarbonate (15 ml). Each aqueous layer was extracted with dichloromethane (2 × 20 ml). The combined organic phases were washed with brine (20 ml), dried (MgSO_4_), filtered, and concentrated under reduced pressure to afford crude compound **7**. The compound was purified by column chromatography (chloroform:methanol 9:1 v/v) to afford title compound **7** (342 mg) as an orange solid with a good 96.9% yield; mp 220 °C (decomp.); ^1^H NMR (DMSO-D_6_, 600 MHz): *δ* = 11.51 (1H, s, NH), 10.12 (1H, s, NH), 8.75 (1H, br s, ArH), 8.39–8.38 (1H, m, ArH), 8.03–8.02 (1H, m, Ar–H), 7.81–7.76 (2H, m, ArH), 7.73–7.71 (1H, m, ArH), 7.56–7.53 (1H, m, ArH), 4.86 (2H, m, H-12), 3.58 (2H, m, H-13), 3.18 (3H, s, Ar–CH_3_), 2.92 (6H, s, N(CH_3_)_2_), 1.55 (9H, s, *t*-Bu), 1.40 (9H, s, *t*-Bu); ^13^C NMR (DMSO-D_6_, 150 MHz): *δ* = 162.7 (C, HNC(NH)_2_), 162.2 (C, C-11), 153.5 (C, NHCO-*t*-Bu), 152.1(C, NCO-*t*-Bu), 151.6 (C, C-5a), 145.6 (C, C-6a), 139.6 (CH, C-3), 138.4 (C, C-9), 129.9 (CH, C-8), 129.0 (CH, C-1), 127.5 (CH, C-2), 124.6 (C, C-11a), 123.9 (C, C-10a), 123.8 (CH, C-10), 123.0 (CH, C-4), 119.1 (C, C-10b), 115.7 (CH, C-7), 83.3 (C, *t*-Bu), 78.5 (C, *t*-Bu), 54.6 (CH_2_, C-13), 42.7 (CH_2_, C-12), 38.7 (CH_3_, C-14), 36.5 (CH_3_, C-14), 30.7 (CH_3_, *t*-Bu), 27.8 (CH_3_, *t*-Bu), 27.6 (CH_3_, *t*-Bu), 14.5 (CH_3_, Ar–CH_3_); The ^1^H NMR and ^13^C NMR data obtained were in good agreement with that reported in the literature (Sidoryk et al. 2015). HRESIMS *m*/*z*: 561.3189 (C_31_H_41_N_6_O_4_) [M + H]^+^ calc. 561.3179.

##### *N*-guanyl-*N*-[6-(2-dimethylaminoethyl)-11-methyl-6*H*-indolo[2,3-b]quinolin-9-yl]-amine tetrahydrochloride (**3**)

Product **7** (200 mg, 0.357 mM) was treated with TFA (10 ml) and stirred for 24 h (TLC monitoring). The solution was evaporated to dryness, next HCl/CH_3_OH was added and evaporated to dryness. The procedure was repeated three times. The residue was crystallized from ethyl acetate to afford a yellow solid; yield 140 mg (77%); mp 200–202 °C; ^1^H NMR (D_2_O, 500 MHz): *δ* = 8.22–8.21 (1H, m, H-1), 8.18 (1H, d, *J* = 4 Hz, H-10), 8.07–8.05 (1H, m, H-4), 7.92–7.89 (1H, m, H-3), 7.76–7.71(2H, m, H-7, H-8), 7.65–7.62 (1H, m, H-2), 4.76 (2H, m, H-12), 3.68 (2H, m, H-13), 3.09 (6H, s, N(CH_3_)_2_), 2.96 (3H, s, Ar–CH_3_); ^13^C NMR (D_2_O, 125 MHz): *δ* = 159.5 (C, NC(NH)_2_), 150.7 (C, C-11), 149.6 (C, C-5a), 141.5 (C, C-6a), 140.7 (C, C-4a), 135.1 (CH, C-3), 131.8 (C, C-9), 129.9 (CH, C-8), 128.2 (C, C-2), 127.9 (C, C-1), 125.4 (C, C-11a), 124.9 (CH, C-10), 124.3 (C, C-10a), 124.1 (CH, C-4), 119.6 (C, C-10b), 113.6 (CH, C-7), 56.5 (CH_2_, C-13), 46.1 (CH_3_, N(CH_3_)_2_), 40.2 (CH_2_, C-12), 18.0 (CH_3_, Ar–CH_3_); The ^1^H NMR and ^13^C NMR data obtained were in good agreement with that reported in the literature (Sidoryk et al. 2015); ESIMS *m*/*z*: 361.4 (C_21_H_24_N_6_) [M + H]^+^ calcd. 360.4; anal. calcd. for C_21_H_24_N_6_ × 5H_2_O × 4HCl [596.37]: C, 42.29; H, 6.42; N, 14.09; Cl, 23.78 found: C, 42.20; H, 6.59; N, 14.00; Cl, 24.06. HPLC: 96.5%.

### Biological studies

#### In vitro study

Cell lines were purchased from American Type Culture Collection (ATCC). The cell lines A549 and NCI-H460 were maintained in RPMI-1640 (Gibco), HepG2, ACHN and CCD-11Lu were maintained in minimum essential medium (Gibco), HCT116 and HT29 were maintained in McCoy’s Medium (Gibco), and A431 was maintained in Dulbecco’s minimum essential medium. MCF7 was maintained in Minimum Essential Medium (Gibco) with 0.01 mg/ml human insulin (Sigma). All culture media were supplemented with 10% fetal bovine serum (Gibco), streptomycin, and penicillin (Biowest). The cells were grown in standard conditions: 37 °C, 5% CO_2_, 95% humidity, and passaged with 0.25% trypsin-EDTA, until ~80% confluence was reached. During the course of the experiment, the microbiological purity of all cell lines was tested with the Universal Mycoplasma Detection Kit (ATCC).

The cells were seeded in 96-well cell culture plates and on the next day treated with different concentrations of the tested compounds for the next 72 h. The cells were then incubated with 3-(4,5-dimethylthiazol-2-yl)-2,5-diphenyltetrazolium bromide at 37 °C for 3 h. Finally, the medium with the MTT solution was removed and formazan crystals were dissolved by adding DMSO. After mixing, the absorbency was measured at the 570 nm wavelength (690 nm reference filter) using a Microplate Reader EON (BioTek). The experiments were performed in triplicate.

#### In vivo study

##### Mice

Female, 7–8-week-old Balb/c mice and 16-week-old DBA/2 mice were supplied by Mossakowski Medical Research Centre Polish Academy of Sciences (Warsaw, Poland). The mice were maintained in specific pathogen-free conditions. The experiments were performed according to the Interdisciplinary Principles and Guidelines for the Use of Animals in Research, Marketing, and Education issued by the New York Academy of Sciences’ Ad Hoc Committee on Animal Research and approved by the Local Committee for Experiments with the Use of Laboratory Animals, Wroclaw, Poland.

##### Cell lines

Murine mammary gland carcinoma 4T1 and murine lung carcinoma KLN205 cell lines were used and maintained under standard cell culture conditions (humidified atmosphere and 5% CO_2_ at 37 °C). These cell lines were obtained from the ATCC (Rockville, Maryland, USA) and maintained at the Hirszfeld Institute of Immunology and Experimental Therapy (IIET), Wroclaw, Poland.

KLN205 cells were cultured in Eagle’s minimal essential medium (IIET, Wroclaw, Poland) supplemented with 10% fetal bovine serum (GE Healthcare Life Sciences, Utah, USA), 2 mM l-glutamine, 1 mM sodium pyruvate, 1% of MEM nonessential amino acid solution 100x, 100 μg/ml streptomycin (all Sigma-Aldrich, Germany), and 100 IU/ml penicillin (Polfa Tarchomin S.A., Poland).

4T1 cells were cultured in RPMI 1640 w/GlutaMAX medium (Gibco) supplemented with 10% fetal bovine serum (GE Healthcare Life Sciences, Utah, USA), 1 mM sodium pyruvate, 3.5 g/l glucose, 100 μg/ml streptomycin (all Sigma Aldrich, Germany), and 100 IU/ml penicillin (Polfa Tarchomin S.A., Poland).

##### Maximum tolerated dose (MTD)

In order to determine MTD, compound **1** was injected intraperitoneally (i.p.) into a healthy female BALB/c mouse (three mice per dose) at the 2.5, 5, 10, and 15 mg doses of the drug per kg of the body weight, five times a week for 2–3 weeks. The mice body weight was monitored every day and body weight changes were calculated. At the end of the study, the mice were killed and subjected to autopsy.

##### Anticancer activity in the 4T1 mammary gland carcinoma model

Female Balb/c mice were inoculated *orthotopically (ort.)* with 50 µl PBS suspension of the 4T1 cells (2 × 10^5^ cells/mouse) derived from an in vitro culture. On the 7th day after inoculation, when the tumors reached about 45 mm^3^, the mice were divided into groups (*n* = 10) and compound administration started. The compounds were administered intraperitoneally (i.p), compound **1** five times a week at the 5 and 10 mg/kg b.w. doses and cyclophosphamide three times a week at the dose of 25 mg/kg b.w. (all for 3 weeks). The control mice received i.p. aqua pro injection (0.1 ml/10 g b.w.) in the same schedule as for compound **1**.

Tumor dimensions were measured using a caliper, and the body weight of the animals was monitored three times a week. Tumor volume (mm^3^) was calculated using the formula (*a* ^2^ x *b*)/2, where *a* = shortest tumor diameter in millimeters and *b* = longest tumor diameter in millimeters. Tumor growth inhibition for each day was calculated using the formula TGI (%) = − ([(V_t_/V_ctrl_) × 100]−100%), where V_t_ is the mean tumor volume of the treated mice and V_ctrl_ that of the untreated control animals. On the 29th day, all mice were killed and whole blood was collected for the morphological analysis using the Mythic 18 analyzer (PZ Cormay, PolandOrphee). Additionally, spleens, livers, kidneys, and lungs were dissected and weighed. Lungs were further fixed in 4% (v/v) paraformaldehyde in the PBS solution, and the metastatic nodes were counted.

##### Anticancer activity in the murine lung carcinoma KLN205 model

Female DBA/2 mice were inoculated *subcutaneously* (s.c.) with the KLN205 cells (8.5 × 10^5^ cells/mouse) derived from an in vitro culture. On the 7th day after the inoculation, when tumors reached about 60 mm^3^, the mice were divided into groups (*n* = 7–8) and compound administration started. The compounds were administered intraperitoneally (i.p): compound **1** five times a week at the 10 and 20 mg/kg b.w. doses and cyclophosphamide once a week at the dose of 100 mg/kg b.w. (all for 2.5 weeks). The control mice received i.p. aqua pro injection (0.1 ml/10 g b.w.) in the same schedule as for compound **1**.

Tumor dimensions were measured using a caliper, and the body weight of the animals was monitored three times a week. Tumor volume (mm^3^) was calculated using the formula described above. On day 25, all mice were killed and whole blood was collected for the morphological analysis using the Mythic 18 analyzer (Orphee). The whole blood was further centrifuged (2000x*g*, 4 °C, 15 min) for plasma and analyzed for biochemical parameters (alanine aminotransferase (ALT), aspartate aminotransferase (AST), lactate dehydrogenase (LDH), urea and creatinine (CREA)) using the Cobas c111 analyzer (Roche). Additionally, livers and kidneys were dissected and weighed.

##### MTD of compound **1** determination in BALB/c mice

Compound **1** was injected i.p. at the doses of 2.5, 5, 10, and 15 mg per kg body weight, five times a week for 2–3 weeks. No toxicity of the 2.5 mg/kg b.w. dose was observed. Doses 5 and 10 mg/kg b.w. had low toxicity for the Balb/c mice (max. body weight loss reached 7 and 9.5%, respectively). Compound **1** at the dose of 15 mg/kg b.w. was toxic for the Balb/c mice. Max. body weight loss reached 21% and one mice died.

## Results

### Studies on the efficient methods for the construction of guanidine-substituted indolo[2,3-b]quinolines

**Scheme 1:**
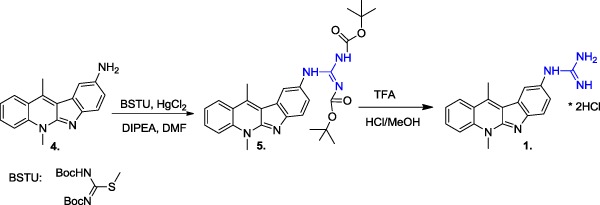
The standard synthesis method of the indolo[2,3-b]quinoline guanidine derivative

As it had been shown in our previous studies (Sidoryk et al. 2015), the presence of the guanidine group is a very important element affecting the cytotoxicity and selectivity of the indolo[2,3-b]quinoline guanidine derivatives. Until now the indolo[2,3-b]quinoline guanidine derivative (**1**) has been prepared in the following established sequence: the amidination of indolo[2,3-b]quinoline-amine (**4**) using BSTU (*N*,*N*’-Bis(tert-butyloxycarbonyl)-*S*-methylisothiourea) as the guanylation reagent and the removal of Boc protection groups with trifluoroacetic acid (Scheme 1).

The limitations of this approach include a relatively low yield of amidination (50–60%) and the release of gaseous methanethiol (CH_3_SH) with a distinctive stench during the reaction. Thus, we have decided to search for more efficient synthetic methods to prepare compounds **1** and **3**.

**Scheme 2:**
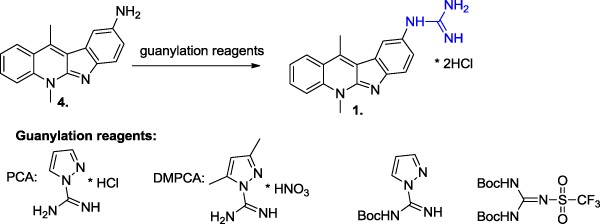
The reagents used for the optimization of compound **4** guanidylation process

After a detailed literature search, numerous reagents leading to obtain different guanylate compounds have been found. Common guanidylation methods use mono-, di-Boc, or mono-and di-Z-derivatives of 1*H*-pyrazole-1-carboxamidine (Bernatowicz et al. 1992; Massimba-Dibama et al. 2015). However, the PCA reagent (1*H*-pyrazole-1-carboxamidine) seemed to be the most favorable, because it enables the introduction of the guanidine group in a single step and does not require the removal of any protecting group. This may increase the overall yield of the entire process and eliminate the problem of gaseous thiol released during the BSTU guanidylation. We have started to test commercially available reagents and literature procedures using PCA (1*H*-pyrazole-1-carboxamidine hydrochloride), DMPCA (3,5-dimethyl-1-pyrazolylformaminidium nitrate), *N*-Boc-1H-pyrazole-1-carboxamidine, and 1,3-di-Boc-2-(trifluoromethylsulfonyl)guanidine (Scheme 2) in the guanylation of **4** (Baker et al. 2004). The progress of the reactions was monitored by TLC methods. For the PCA or DMPCA reagents, HPLC methods were applied to examine **1** formation.

While using the PCA or DMPCA as guanylation reagents, a prolonged reaction time was essential to the consumption of the starting material **4** (Table 1). For both reagents, the minimum reaction time was determined as 10 days, although even after that time the traces of substrate **4** were observed in the reaction mixtures. Moreover, the HPLC analysis revealed the presence of a large number of unknown impurities in the mixture, especially for DMPCA, when only 44.7% of the expected product **1** was observed. The long reaction time and the tendency to form various impurities eliminated PCA and DMPCA procedures as ineffective for the synthesis of indolo[2,3-b]quinoline guanidine derivatives. *N*-Boc-1*H*-pyrazole-1-carboxamidine was the next examined reagent. In standard conditions, at ambient temperature, the reaction progress was not observed in time. Although we prolonged the reaction time to 5 days and increased the temperature to 50–60 °C, only unchanged substrate **4** was detected in the TLC method.

**Table 1:** The results of the guanidylation process of **4** according to the HPLC methods

Guanylation reagent	Reaction time	HPLC^a^		
		Substrate **4** (Rt = 17.0) (%)	Product **1** (Rt = 19.1) (%)	Intermediate **5** (Rt = 30.8)
PCA	24 h	75.5	18.5	–
	48 h	51.8	38.5	–
	72 h	34.2	54.5	–
	10 days	15.5	67.2	–
	14 days	11.9	**68.3**	–
DMPCA	24 h	81.7	7.5	–
	48 h	64.8	19.5	–
	72 h	49.9	30.4	–
	10 days	16.5	**44.7**	–
*N*-Boc-1*H*-pyrazole-1-carboxamidine	48 h		– ^b^	–
*N*,*N*’-di-Boc-*N*′′-triflylguanidine	5 h	–	**97.38**	94.82%

^a^ HPLC methods were described in the Experimental section

^b^ According to the TLC method

Bold values are the best result observed in the experiment

**Fig. 2:**
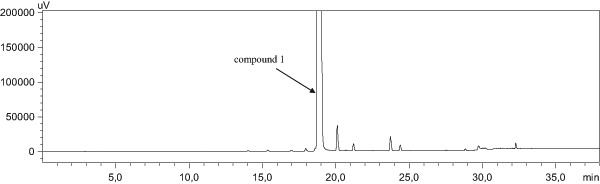
Chromatogram of compound **1** obtained with an HPLC method described in the Experimental Section

**Scheme 3:**
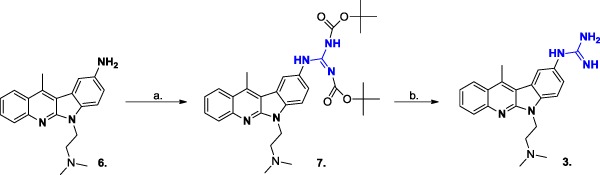
Reagents and conditions: **a** 1,3-di-Boc-2-(trifluoromethylsulfonyl) guanidine, DIPEA, DCM, rt. **b** TFA, HCl/MeOH

During our studies, the best result was achieved when a completely protected guanidine derivative 1,3-di-Boc-2-(trifluoromethylsulfonyl)guanidine (*N*,*N*′-di-Boc-*N*″-triflylguanidine) was applied as a guanylation reagent. The guanylation method with *N*,*N*′-di-Boc-*N*″-triflylguanidine seems to be the most efficient for the guanidylation of amines. Thus, it is commonly applied as the method of choice for the reaction carried out in a solution or on solid phase (Feichtinger et al. 1998a, b). Generally, guanidylations with pyrazole-1-carboxamidines and *S*-alkylisothioureas are kinetically slower in comparison to that using *N*,*N*′-di-Boc-*N*″-triflylguanidine. Moreover, simple preparation of *N*,*N*′-di-Boc-*N*″-triflylguanidine from a commercially available source is also conceivable, which makes this guanidylation reagent very attractive for the chemists (Baker and Goodman 1999). The process of guanidylation of **4** through *N*,*N*′-di-Boc-*N*″-triflylguanidine was performed similarly to the known procedures (Magri et al. 2015), i.e., in DCM, in the presence of DIPEA, at room temperature. It was monitored by TLC and finally HPLC methods. The total consumption of **4** was observed after 5 h with the yield of guanidylation after work-up equal to 95%. The purity of intermediate **5** was characterized as 94.82% using HPLC. The treatment of the protected Boc-derivative (**5**) with trifluoroacetic acid and then with HCl/MeOH gave *N*-guanyl-*N*-(5,11-dimethyl-5*H*-indolo[2,3-b]chinolin-9-yl)-amine dihydrochloride **1** with a good 84% yield and 97.38% purity (Fig. 2). Taking into consideration the above results, a similar strategy was applied for the synthesis of guanidine derivatives of 6*H*-indolo[2,3-b]quinoline (**3**). Compound **7** was obtained in the guanidylation process with a 96.9% yield (Scheme 3). The Boc-removal of compound **7** with trifluoroacetic acid and then the treatment of the residue by hydrogen chloride in methanol gave the hydrochloride derivatives of 6*H*-indolo[2,3-b]quinoline, compound **3**, with a 77% yield. The purity of the obtained final products **1** and **3** was proved by the C-18 RPHPLC method using acetonitrile–water as the mobile phase.

### Biological studies

#### In vitro study

The synthesized compounds **1**, **3** and the reference compound DiMIQ were evaluated for their in vitro antiproliferative activity against human lung cancer (A549), human lung cancer (H460), human colorectal cancer (HCT 116), human colorectal cancer (HT-29), human kidney cancer (ACHN), human hepatocellular cancer (HepG2), human breast cancer (MCF7), human skin cancer (A431), and human lung fibroblasts (CCD11Lu) cell lines.

**Fig. 3:**
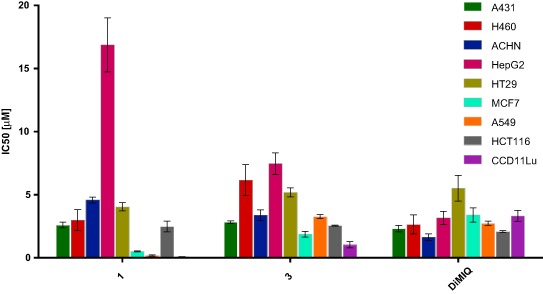
Comparison of the antiproliferative activity (IC_50_) of the indolo[2,3-b]quinoline derivatives **1**, **3**, and DiMIQ

All three tested compounds showed cytotoxic properties (Fig. 3). However, compound **3** seemed to be significantly weaker than **1** and the DiMIQ reference compound. The most important differences in the activity against specific types of human cell lines were observed for compound **1**. The MCF7 breast and A549 lung carcinoma cells turned out to be the most sensitive to **1**, with IC_50_ = 0.5 µM and 0.19 µM, respectively. These results appeared to be 5–15 times higher in comparison to that collected for DiMIQ (IC_50_ = 3.4 and 2.74 µM). For two investigated cell lines, i.e., ACHN derived from human kidney cancer and HepG2 derived from human liver cancer, compound **1** exhibited the weakest cytotoxic properties among all studied compounds, while DiMIQ was the most active. These visible differences seem to be noteworthy and would present an interesting area for further scientific investigation in order to find specific biomarkers influencing sensitivity to particular cell lines. The activities of compounds **1** and DiMIQ against other investigated cell lines were comparable on the level of a few micromoles. Despite displaying high cytotoxicity against normal cells (CCD11Lu), compound **1** was selected for extended in vivo studies because of its interesting in vitro profile against breast and lung cancer cell lines.

#### In vivo study

Compound **1** and cyclophosphamide (Endoxan, Baxter) were dissolved in aqua pro injection prior to use.

##### Anticancer activity of compound **1** in the 4T1 mammary gland carcinoma model

**Fig. 4:**
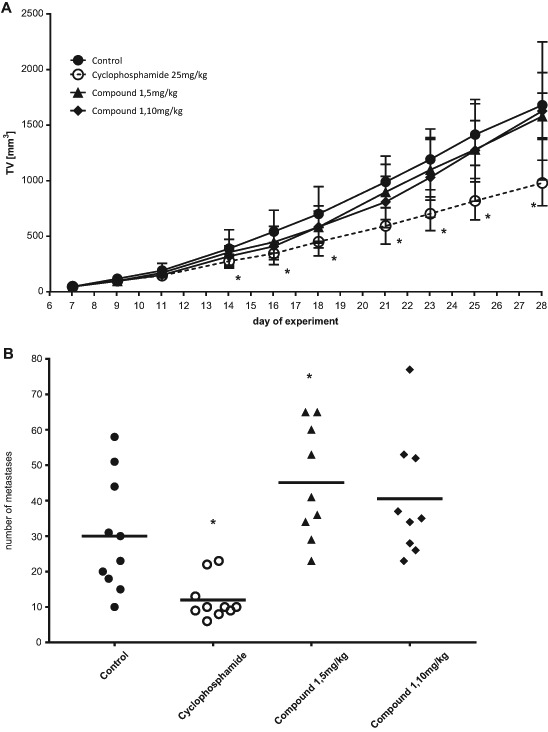
Kinetics of the 4T1 tumor growth (**a**) and number of metastatic nodes in lungs (**b**) in Balb/c mice treated with compound **1**. Compound **1** was administered IP at the doses of 5 and 10 mg/kg b.w. five times a week; cyclophosphamide was administered IP at the dose of 25 mg/kg b.w. three times a week; *asterisk* indicates the statistical significance vs the control group (*p* < 0.05; Mann–Whitney U test, Statistica 10.1)

Female Balb/c mice were inoculated *orthotopically (ort.)* with the 4T1 cells (2 × 10^5^ cells/mouse). On the 7th day after cell inoculation, compounds' administration began (intraperitoneally). Compound **1** was administered five times a week at the strength of 5 and 10 mg/kg b.w. The anticancer activity of compound **1** was not observed (Fig. 4a), only cyclophosphamide at the dose of 25 mg/kg b.w (three times a week) inhibited 4T1 tumor growth by 30–42%. At the end of the experiment (day 29), all mice were killed. Whole blood was collected for the morphological analysis and spleens, livers, kidneys, and lungs were dissected and weighed. Additionally, metastatic nodes in the lungs were counted. Compound **1** increased the number of tumor metastasis in the lungs (Fig. 4b), but did not influence the lungs’ weight (Table 2). Cyclophosphamide diminished the number of metastatic nodes in lungs by 60% (Fig. 4b), and also the weight of lungs (Table 2) was lower in comparison to that in the control mice.

**Table 2:** The influence of compound **1** on the blood morphology and organ weight in 4T1 tumor-bearing mice

	Control	Cyclophosphamide	Compound** 1**	Compound** 1**
		25 (mg/kg)	5 (mg/kg)	10 (mg/kg)
WBC (10^3^/µl)	232.6 ± 64.7	244.2 ± 82.2	298.9 ± 78.9	342.4 ± 110.6*
LIMF (10^3^/µl)	22.8 ± 5.3	18.9 ± 3.9	33.6 ± 6.1*	36.7 ± 8.9*
MON (10^3^/µl)	11.4 ± 3	14.9 ± 4.1	23.9 ± 7.7*	29.7 ± 10.4*
GRAN (10^3^/µl)	198.3 ± 60.1	210.4 ± 75.0	241.3 ± 67.3	276.0 ± 92.6
LIMF (%)	10.2 ± 2.4	8.1 ± 1.5	11.6 ± 2.1	11.0 ± 1.4
MON (%)	5.0 ± 0.4	6.4 ± 1.3*	7.9 ± 0.8*	8.7 ± 1.0*
GRAN (%)	84.8 ± 2.6	85.5 ± 2.7	80.4 ± 2.1*	80.3 ± 2.1*
Erythrocytes (10^6^/µl)	8.9 ± 0.7	9.4 ± 0.8	9.1 ± 0.6	9.6 ± 1.3
hemoglobin (g/dl)	15.6 ± 1.4	17.0 ± 1.3	16.4 ± 1.2	17.5 ± 2.4
hematocrit (%)	42.7 ± 4.4	47.5 ± 3.4*	45.4 ± 3.0	49.4 ± 6.8*
MCV (fL)	47.9 ± 1.5	50.5 ± 2.3*	50.1 ± 1.8*	51.2 ± 2.1*
MCH (pg)	17.5 ± 0.7	18.1 ± 0.4	18.1 ± 0.4	18.1 ± 0.3*
MCHC (g/dl)	36.7 ± 1.5	35.8 ± 1.4	36.2 ± 1.6	35.5 ± 1.4
RDW (%)	16.5 ± 0.4	18.4 ± 1.4*	16.4 ± 1.0	16.5 ± 0.9
platelets (10^3^/µL)	618.7 ± 146.7	852.4 ± 126.3*	745.3 ± 109.8	737.8 ± 260.7
Liver (g)	1.150 ± 0.1	1.214 ± 0.17	1.187 ± 0.14	1.230 ± 0.22
Spleen (g)	0.809 ± 0.13	0.814 ± 0.22	0.703 ± 0.07*	0.731 ± 0.12
Kidneys (g)	0.265 ± 0.03	0.278 ± 0.03	0.270 ± 0.03	0.258 ± 0.04
Lung (g)	0.325 ± 0.1	0.228 ± 0.03*	0.330 ± 0.08	0.327 ± 0.08

Control and groups treated with cyclophosphamide at the dose of 25 mg/kg (3 t/w) and compound **1** at the doses of 5 and 10 mg/kg (5 t/w)

* Statistical significance vs. the control group, (*p * <  0.05; Mann-Whitney U test, Statistica 10.1)

The overall condition of the mice bodies was monitored, and no body loss was observed (data not shown). In groups that received compound **1,** the weight of the liver and kidneys was similar to the control groups (Table 2). No changes in the number of erythrocytes and in the level of hemoglobin were observed in the treated groups (Table 2). After the treatment with compound **1** or cyclophosphamide, increased levels of hematocrit, MCV and number of platelets were observed. Compound **1** increased the number of leukocytes (WBC), but the percent of monocytes was higher and the percent of granulocytes was lower than that in the control mice (Table 2).

##### Anticancer activity of compound **1** in the KLN205 murine lung carcinoma model

**Fig. 5:**
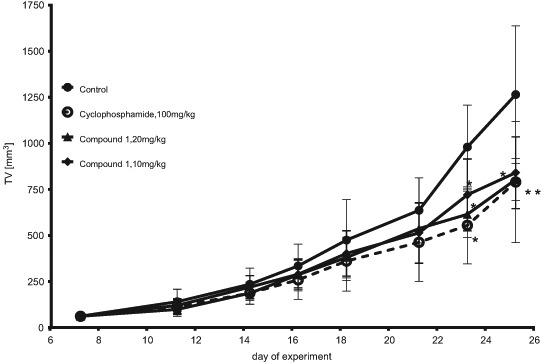
Kinetics of the KLN205 tumor growth in DBA/2 mice treated with compound **1**. Compound **1** was administered IP at the doses of 10 and 20 mg/kg b.w. five times a week; cyclophosphamide was administered IP at the dose of 100 mg/kg b.w. once a week; *asterisk* indicates the statistical significance vs. the control group (*p* < 0.05; Mann-Whitney U test, Statistica 10.1)

**Fig. 6:**
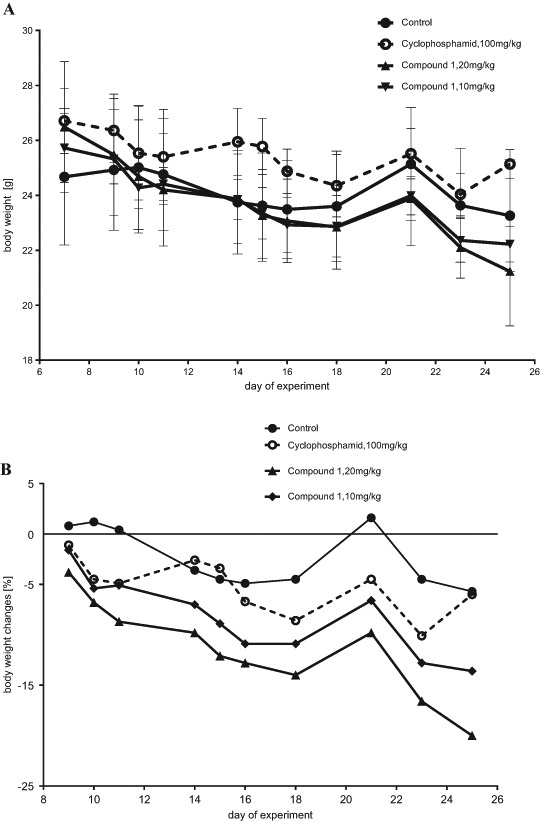
Body weight (**a**) and body weight changes (**b**) in DBA/2 mice bearing KLN205 tumors treated with compound **1**. Compound **1** was administered IP at the doses of 10 and 20 mg/kg b.w. five times a week; cyclophosphamide was administered IP at the dose of 100 mg/kg b.w. once a week

Compound **1** was administered five times a week at the strength of 10 and 20 mg/kg b.w. starting from the 7th day after the KLN205 cells' inoculation. The anticancer activity of compound **1** was observed at the end of the experiment (Fig. 5), tumor growth inhibition reached 27–34% at the dose of 10 mg/kg and about 37% at the dose of 20 mg/kg. Toxic effects of compound **1** were observed in tumor-bearing DBA/2 mice. Body weight loss (Fig. 6b) was 9–14% at the 10 mg/kg dose and 10–20% at the 20 mg/kg dose (one mouse died on the last day of the experiment). Compound **1** decreased the number of erythrocytes as well as the levels of hemoglobin, hematocrit (which may demonstrate side effect of the treatment, such as anemia), and creatinine (CRE) in the blood in comparison to the control group (Table 3). The percentage of lymphocytes increased and the percentage of granulocytes decreased, but the total number of leukocytes was similar to that of the control group. Diminished liver weight in the group that received compound **1** at the 20 mg/kg dose was observed (Table 3). Cyclophosphamide used as a positive control at the 100 mg/kg b.w. dose (once a week) inhibited tumor growth by 27–43% (Fig. 5) and slightly decreased body weight (7–10%). Side effects of the treatment, such as a decrease in the number of erythrocytes and leukocytes (WBC) (an increased percentage of lymphocytes and a decreased percentage of monocytes and granulocytes was observed) were also observed. The cyclophosphamide treatment also diminished the levels of hemoglobin, hematocrit, CRE, as well as alanine aminotransferase (ALT), aspartate aminotransferase (AST), and lactate dehydrogenase (LDH) in the blood. Liver weight was higher in this group.

**Table 3:** Blood morphology, biochemistry, and organ weight in mice bearing KLN205 tumors

	**Control**	Cyclophosphamide	Compound** 1**	Compound** 1**
		100 (mg/kg)	10 (mg/kg)	20 (mg/kg)
WBC (10^3^/µl)	33.2 ± 6.0	3.5 ± 0.8*	37.5 ± 17.4	27.8 ± 17.5
LIMF (10^3^/µl)	15.0 ± 6.0	2.4 ± 0.7*	24.5 ± 16.2	17.4 ± 15.2
MON (10^3^/µl)	3.5 ± 0.8	0.3 ± 0.1*	3.0 ± 0.5	2.4 ± 0.6*
GRAN (10^3^/µl)	14.7 ± 2.8	0.8 ± 0.2*	10.0 ± 2.1*	8.0 ± 2.5*
LIMF (%)	44.4 ± 11.8	68.3 ± 5.4*	59.7 ± 15.0	54.2 ± 19.4
MON (%)	10.7 ± 2.5	8.0 ± 1.2*	9.6 ± 4.3	10.9 ± 5.3
GRAN (%)	45.0 ± 9.5	23.7 ± 4.2*	30.7 ± 10.8*	34.9 ± 14.1
Erythrocytes (10^6^/µl)	7.3 ± 1.1	5.9 ± 0.7*	4.7 ± 1.3*	5.8 ± 1.5
hemoglobin (g/dl)	11.4 ± 1.2	9.4 ± 0.9*	8.4 ± 2.1*	10.1 ± 1.8
hematocrit (%)	31.4 ± 3.0	25.0 ± 2.6*	24.1 ± 5.1*	27.7 ± 4.0
MCV (fL)	43.5 ± 4.6	42.7 ± 2.6	51.9 ± 5.4	49.7 ± 7.3
MCH (pg)	15.8 ± 1.4	16.1 ± 0.8	17.9 ± 1.1	17.9 ± 1.9
MCHC (g/dl)	36.4 ± 1.0	37.7 ± 0.6	34.5 ± 1.8	36.3 ± 1.3
RDW (%)	22.0 ± 2.6	22.2 ± 2.0	24.3 ± 2.5	26.5 ± 3.1
platelets (10^3^/µl)	817.3 ± 67.7	1477.0 ± 97.7*	750.3 ± 125.7	942.8 ± 38.3
ALT (U/l)	29.6 ± 3.9	23.0 ± 1.2*	25.6 ± 2.5*	24.8 ± 1.6*
AST (U/l)	98.1 ± 11.2	66.6 ± 8.6*	86.8 ± 9.7*	95.1 ± 12.1
LDH (U/l)	429.7 ± 67.6	325.5 ± 62.0*	406.4 ± 61.9	457.1 ± 85.6
CRE (umol/l)	6.3 ± 1.2	3.1 ± 0.8*	5.6 ± 1.9	4.4 ± 1.1*
urea (mmol/l)	6.2 ± 1.6	6.1 ± 1.1	7.0 ± 1.8	6.2 ± 1.1
Liver (g)	1.473 ± 0.09	1.666 ± 0.11*	1.384 ± 0.11	1.275 ± 0.16*
Kidneys (g)	0.344 ± 0.04	0.366 ± 0.02	0.327 ± 0.04	0.328 ± 0.04

Control and groups treated by cyclophosphamide at the dose of 100 mg/kg (1 t/w) or compound **1** at the doses of 10 and 20 mg/kg (5 t/w)

* Statistical significance vs. the control group, (*p*  <  0.05; Mann–Whitney U test, Statistica 10.1)

## Discussion

During our previous studies it had been demonstrated that the guanidylation by *N*,*N*’-Bis(tert-butyloxycarbonyl)-*S*-methylisothiourea allows to obtain samples of indolo[2,3-b]quinoline guanidine derivatives for biological screening in average yields. However, this approach seemed to be problematic in the production of gram samples of compounds because of the mephitic methyl mercaptan release. In the present paper an optimization of the guanidylation process in order to obtain the guanidine derivatives of indolo[2,3-b]quinoline has been discussed. The effectiveness of the guanylation using common guanylation reagents, such as 1*H*-pyrazole-1-carboxamidine hydrochloride, 3,5-dimethyl-1-pyrazolylformaminidium nitrate, *N*-Boc-1*H*-pyrazole-1-carboxamidine, and 1,3-di-Boc-2-(trifluoromethylsulfonyl)guanidine (*N*,*N*′-di-Boc-*N*″-triflylguanidine), was verified. As a result of our studies, a high-yield procedure with *N*,*N*’-di-Boc-*N*″-triflylguanidine was applied to synthesize *N*-guanyl-*N*-(5,11-dimethyl-5*H*-indolo[2,3-b]qinolin-9-yl)-amine dihydrochloride **1** which had been selected for specific in vitro and in vivo biological research. Compound **1** was obtained in a gram scale in the presence of *N*,*N*’-di-Boc-*N*″-triflylguanidine reagent with the overall yield of 80 and 97.38% purity according to the HPLC method. Then, a comprehensive in vitro study on the antiproliferative activity of DiMIQ, the reference compound, and two guanidine analogs **1** and **3** against eight cancer cell lines was completed. The highest activity was shown by compound **1** against A549 and MCF7 lines with the IC_50_ values equal to 0.19 µM and 0.5 µM, respectively. Therefore, compound **1** was selected for further investigations in the in vivo mouse models of 4T1 mammary gland carcinoma and KLN205 murine lung carcinoma. As far as the 4T1 model is concerned, we did not observe any anticancer activity of **1** at the 5 and 10 mg/kg b.w. doses, whereas the anticancer effect was observed in the KLN205 model. The tumor growth inhibition induced by compound **1** reached 37% at the 20 mg/kg dose, which was comparable to that of cyclophosphamide used as a positive control. This commonly used therapeutic agent inhibited tumor growth in mice by 27–43% at the dose of 100 mg/kg. Moreover, biochemistry research based on monitoring blood parameters such as alanine aminotransferase ALT, aspartate aminotransferase AST, lactate dehydrogenase LDH, and urea and creatinine CREA was performed. Compound **1** decreased the number of erythrocytes and the levels of hemoglobin, hematocrit, as well as creatinine in the blood in comparison to the control group. Although the total number of leukocytes was similar to that in the control group, an increase in lymphocytes and a decrease in granulocytes were observed. A similar effect was measured for cyclophosphamide.

Although cancer cell lines have a prominent role in the initial stages of drug discovery, it was found that even cancer-specific drugs do not show higher efficacies in the cell lines representing respective tissues (Jaeger et al. 2015). Thus, we verified the results of our enhanced in vivo studies in two mouse models, which led us to the conclusion that compound **1** displayed an anticancer activity in the KLN205 mouse model. This result qualified guanidine derivative **1**, which is an effective inducer of apoptosis and does not induce hemolysis (Sidoryk et al. 2015), for further investigations in the cell line xentograft models CLX and/or patient derived tumor xenografts (PDTX) which resemble tumors growing in a patient.

Although the core structure of DiMIQ meets all the criteria for the DNA intercalation and TOPO2 inhibition, previous studies had shown that TOPO2 may not be the main cellular target for different DiMIQ analog compounds. Therefore, for the conjugates containing the guanidine group, a study of biological interactions with the tumor models in order to find the selectivity factors related to specific signaling pathways responsible for the cancer growth is intended.

## Conclusions

Summarizing, an efficient of the guanidylation process for obtaining guanidine derivatives of indolo[2,3-b]quinoline **1**, using *N*,*N*’-di-Boc-*N*″-triflylguanidine has been performed. Compound **1**, produced in a gram scale, was next tested for its anticancer activity in vivo. The biological studies proved that **1** has a high anticancer activity against KLN205 murine lung carcinoma with a 37% tumor growth inhibition at the 20 mg/kg dose. These promising results of our conducted research encourage further studies using PDTX cancer models.
